# Water

**Published:** 1866-04

**Authors:** 


### WATER.

As to the greatest number of farms, it is the best plan generally to dig a well or build a cistern on a spot to be covered by the roof of the dwelling; and perhaps, under all the circumstances of the case, the cheapest, most available, and least troublesome method of obtaining the water, is by means of the old-fashioned pump, which does not often get out of order, is easily repaired, and, under the above circumstances, is not likely to freeze.

The roof of almost every farm-house will catch enough water to supply the wants of a family; this is most healthful to drink and is best for cooking purposes, and for washing clothes; and, indeed, for all cleansing purposes. When it is not practicable to have a good cistern, a running spring is next best, and a well next to that; but whether well, cistern, or spring, all should be most thoroughly washed out, scraped, and washed out again in the spring and autumn, especially the latter.

